# Barriers to vaccine acceptance and immunization coverage in Kazakhstan: a mixed-methods study using the COM-B framework

**DOI:** 10.3389/fpubh.2025.1600363

**Published:** 2025-06-17

**Authors:** Lena Kassabekova, Manar Smagul, Gaukhar Nukenova, Aigul Satayeva, Bibigul Aubakirova, Gulnur Zhakhina, Aizhan Yesmagambetova

**Affiliations:** ^1^Scientific and Practical Center for Sanitary and Epidemiological Expertise, Astana, Kazakhstan; ^2^National Center for Public Health, Astana, Kazakhstan; ^3^Nazarbayev University School of Medicine, Astana, Kazakhstan

**Keywords:** vaccine hesitancy, immunization coverage, public health, vaccine acceptance, COM-B framework

## Abstract

**Background:**

Vaccine hesitancy remains a significant public health challenge, affecting immunization coverage and increasing the risk of vaccine-preventable diseases. Understanding the factors influencing vaccine acceptance is crucial for developing targeted interventions. This study examines immunization coverage, vaccine hesitancy, and barriers to vaccine uptake in Kazakhstan, focusing on routine childhood immunization, COVID-19 vaccination, and HPV vaccination.

**Methods:**

A mixed-methods approach was used, combining quantitative immunization coverage data with qualitative insights from focus group discussions (FGDs). Immunization trends were presented using national data from 2020 to 2022. The study included FGDs with healthcare workers, unvaccinated older adults, mothers/caregivers of unvaccinated young children and of adolescent girls, focusing on COVID-19, routine immunization, and HPV vaccination. Thematic analysis was used for defining main themes in discussions.

**Results:**

Although routine immunization coverage improved in 2022 compared to 2020, general vaccine refusals increased by 2.62 times. The primary reasons for vaccine refusal included personal beliefs (68%), concerns about vaccine safety (54%), and distrust in healthcare institutions (39%). COVID-19 vaccine hesitancy was particularly high among older adult individuals, influenced by skepticism regarding vaccine safety, multiple vaccine options, and conspiracy theories. Healthcare professionals exhibited mixed confidence in COVID-19 vaccines expressing concerns about long-term safety. HPV vaccination hesitancy was linked to limited awareness and misconceptions about cervical cancer.

**Conclusion:**

Despite improvements in immunization coverage, vaccine hesitancy remains a critical barrier in Kazakhstan. Addressing misinformation, enhancing healthcare communication, and implementing targeted educational campaigns are essential for improving vaccine acceptance and public trust in immunization programs.

## 1 Introduction

Vaccination is widely regarded as one of the most effective and cost-efficient public health measures, significantly reducing the global burden of vaccine-preventable diseases. However, achieving and maintaining high immunization coverage requires addressing barriers related to vaccine access, acceptance, and trust in healthcare systems (1). Although numerous countries have made significant advancements in routine immunization coverage, challenges such as vaccine hesitancy, misinformation, and logistical constraints continue to hinder progress, particularly with the introduction of new vaccines. For instance, measles – a disease entirely preventable through vaccination – has experienced a fivefold increase globally, largely attributable to vaccine hesitancy (2).

Kazakhstan is currently facing a critical public health challenge, with a fivefold increase in measles cases since 2023, ranking third globally behind Yemen and India despite its comparatively small population (3). Alarmingly, 96% of cases occur among the unvaccinated, primarily children, reflecting a significant decline in vaccination rates driven by disruptions during the COVID-19 pandemic and the rise of a strong anti-vaccination movement (3). A cross-sectional online-based study conducted by Akhmetzhanova et al. revealed that 35% of respondents (*n* = 387) in Kazakhstan displayed hesitancy toward vaccines, with approximately 22% associating vaccination with autism (4). These factors underscore the urgent need for targeted interventions to address vaccine hesitancy and improve immunization coverage.

The COVID-19 pandemic further highlighted the critical role of vaccines in mitigating public health crises, while simultaneously amplifying public concerns and skepticism surrounding vaccine safety, efficacy, and development timelines. According to the results of a cross-sectional study conducted in 2020, more than 36% of surveyed individuals were hesitant about receiving the COVID-19 vaccine (5). Moreover, the same study found that approximately 33% of respondents would not vaccinate their daughters against human papillomavirus (HPV), 50% would not vaccinate their children, and 49% would not vaccinate themselves against seasonal influenza (5). Planned HPV vaccination was officially introduced in Kazakhstan only in 2024 (6). The free, routine voluntary vaccination against HPV is conducted in school institutions among girls aged 11–13, following the signing of informed consent by parents or guardians. For those who do not fall within this age group, the vaccine is available through healthcare facilities, ensuring broader access for the population. Prior to this, an attempt to implement the program in 2013 faced significant resistance, largely fueled by widespread panic and exaggerated media reports about potential side effects (7). This led to the suspension of the program, leaving a gap in efforts to prevent HPV-related diseases.

Given these challenges, understanding public attitudes toward vaccination in Kazakhstan and identifying the barriers that hinder vaccine acceptance have become crucial. The Capability, Opportunity, Motivation, and Behavior (COM-B) model developed by Michie et al. (8), is a theoretical framework used to understand and influence behavior change. It provides a useful framework for addressing these barriers by highlighting the key factors influencing vaccine behavior. By analyzing these components, we can design targeted interventions that improve public knowledge, enhance access to vaccines, and address underlying motivational factors, ultimately fostering higher vaccination rates. The aim of this study is to explore these attitudes and barriers in depth, providing valuable insights to inform strategies for improving vaccination coverage and addressing vaccine hesitancy in the country.

## 2 Materials and methods

### 2.1 Immunization coverage statistics

Immunization data for the population of Kazakhstan from 2020 to 2022 were collected through reports submitted by all polyclinics to the National Center for Public Health of the Ministry of Healthcare of the Republic of Kazakhstan. These data encompass information on mandatory vaccinations. For newborns: Bacillus Calmette-Guérin (BCG), Hepatitis B vaccine (HBV), and Diphtheria-Tetanus-Pertussis vaccine (DTP); for children under 2 years of age: Pneumococcal Conjugate Vaccine (PCV), Oral Poliovirus Vaccine (OPV4), and Measles-Mumps-Rubella vaccine (MMR); revaccination with MMR administered at 6 years of age; and Tetanus-Diphtheria (Td) vaccine administered at 16–17 years of age. These data serve as a comprehensive source for monitoring and evaluating the implementation of the national immunization schedule.

#### 2.1.1 Sampling for focus group discussions

This cross-sectional qualitative study was conducted in Kazakhstan from March 13 to March 31, 2023. The study sites were carefully selected to ensure representativeness and enable comparisons, with data collection taking place in five cities, one city from each of the country’s five regions to ensure representativeness: Almaty, Taldykorgan, Karaganda, Pavlodar, and Oral (Uralsk). To achieve representativeness, focus group discussions (FGDs) were conducted in each city with various target groups. The study included:

1 Ten FDGs with healthcare workers about COVID-19 vaccination, routine immunization and HPV vaccination:

1.1 Five FGDs with physicians, one in each region. These groups included physicians from diverse professional and geographical backgrounds to ensure a representative and heterogeneous sample. Total of 60 participants.
1.2 Five FGDs with nurses, one in each region, following the same principles to capture varied perspectives and ensure inclusivity. Total of 64 participants.

2 Five FGDs with older adults (aged 65 years and older) who had not received COVID-19 vaccination:

2.1 Discussions focused on perceptions, attitudes, and barriers to COVID-19 vaccination. One FGD was conducted in each city to cover all five regions. Total of 46 participants.
3 Five FGDs with mothers or caregivers of unvaccinated young children (aged 2–2.5 years):

3.1 These discussions explored attitudes toward routine immunization. One FGD was conducted in each city. Total of 43 participants.
4 Five FGDs with mothers or caregivers of adolescent girls (aged 9–14 years):

4.1 These discussions focused on HPV vaccination. As with other groups, one FGD was conducted in each city. Total of 53 participants.

This sampling strategy and design ensured comprehensive coverage of perspectives across different demographic and professional groups, contributing to a robust and representative dataset for thematic analysis. Focus group discussions (FGDs) were semi-structured and guided by a set of open-ended prompts designed to explore participants’ vaccine knowledge, attitudes, barriers, and behavioral drivers. Each FGD included 9–13 participants, grouped by stakeholder category. Discussions lasted approximately 60–90 min and were conducted in Kazakh or Russian. All sessions were audio-recorded, transcribed verbatim, and translated into English for analysis. We applied framework analysis, mapping responses onto the COM-B domains (Capability, Opportunity, Motivation–Behavior). Thematic coding was performed by two researchers, and we noted the frequency of specific themes (e.g., “religious beliefs” or “vaccine novelty”) across groups to indicate the salience and recurrence of issues. These details enhance transparency and support the validity of our qualitative findings.

#### 2.1.2 Data collection and analysis

The study was conducted in two brief phases of data collection and analysis. Data were gathered through moderated FGDs, led by trained researchers using a discussion guide developed in collaboration with a World Health Organization (WHO) consultant. Subsequently, the data were analyzed by LK and GN.

The theoretical framework underpinning the study and analysis was the Capability-Opportunity-Motivation-Behavior (COM-B) model (8), adapted to vaccination behavior. This model emphasizes interconnected factors, including knowledge, attitudes, intentions, social support, and ease of access to vaccination services. The COM-B model is chosen due to its comprehensive structure for analyzing behavior change by integrating Capability, Opportunity, and Motivation. This framework allows for the identification of specific, actionable factors influencing vaccine hesitancy and acceptance.

## 3 Results

### 3.1 Immunization coverage statistics

In 2022, immunization coverage in Kazakhstan exhibited notable improvements for several vaccines compared to the preceding years (Table 1). Coverage for the primary DTP series reached 97.1%. Similarly, PCV coverage among children under two years increased to 97.7%, while the coverage for the first dose of the MMR vaccine reached 100%.

**Table 1:** Immunization coverage in Kazakhstan in 2020–2022.

Vaccine	2020	2021	2022
BCG (newborns)	92.8%	93.7%	93.4%
BCG revaccination (6 years)	51.9%	53.3%	57.1%
HBV (newborns)	92.6%	92.4%	93.1%
DTP (under 1 year)	88.3%	94.7%	97.1%
DTP revaccination (18 months)	88.5%	86.9%	94.9%
DTP revaccination (6 years)	91.7%	95.5%	97.0%
PCV (under 2 years)	88.7%	92.6%	97.7%
OPV4 (under 2 years)	91.9%	97.8%	93.1%
MMR (under 2 years)	92.9%	97.4%	100%
MMR revaccination (6 years)	90.7%	95.9%	97.3%
Td (adolescents 16–17 years)	86.7%	94.8%	98.8%
Td (adults)	89.3%	93.2%	95.7%

BCG – Bacille Calmette-Guérin vaccine (for tuberculosis); HBV – Hepatitis B vaccine; DTP – Diphtheria-Tetanus-Pertussis vaccine; PCV – Pneumococcal conjugate vaccine; OPV – Oral polio vaccine; MMR – Measles, Mumps, and Rubella vaccine; Td – Tetanus-Diphtheria toxoid vaccine.

Despite these achievements, certain areas demonstrated challenges. The OPV4 coverage among children under two years dropped to 93.1% in 2022 from 97.8% in 2021. Most of the immunization coverage improvements are observed for Td for adolescents and DTP for children under 1 years of age (Table 1).

An analysis of vaccine refusals revealed a consistent increase in the number of refusals over the three years (Table 2). Total refusals rose from 22,291 in 2020 to 31,414 in 2022, with personal beliefs being the predominant reason. Children under one year constituted most refusal cases, accounting for 67% of the total refusals in 2022. Both religious objections and distrust in vaccines showed a slight decline over the observation period.

**Table 2:** Vaccine refusals in Kazakhstan in 2020–2022 (absolute numbers).

Indicator	2020 (*n* = 22,291)	2021 (*n* = 28,023)	2022 (*n* = 31,414)
New refusals, *n* (%)	5,293 (23)	7,255 (26)	7,145 (23)
Age categories, *n* (%)			
< 1 year old	14,832 (67)	18,345 (66)	21,052 (67)
1–2 years old	3,438 (15)	4,776 (17)	5,034 (16)
3–5 years old	2,506 (11)	2,814 (10)	2,993 (10)
6–15 years old	1,410 (6)	1,761 (6)	1,963 (6)
> = 16 years old	105 (1)	327 (1)	372 (1)
Reasons, *n* (%)			
Personal beliefs	12,703 (57)	16,469 (59)	18,712 (60)
Religious reasons	5,212 (23)	6,356 (23)	7,239 (22)
Distrust in vaccines	2,979 (13)	3,596 (13)	3,710 (12)
Influence of media	1,379 (7)	1,579 (5)	1,731 (6)

### 3.2 Experiences of COVID-19 vaccination

#### 3.2.1 Group 1: healthcare professionals

Healthcare professionals exhibited varying levels of knowledge and confidence in COVID-19 vaccines. While some nurses received training on vaccine safety and efficacy, others reported conflicting information. Physicians relied on manufacturer guidelines and Ministry of Health materials, but language barriers and complex terminology hindered communication with patients.

Concerns included rapid vaccine development, potential long-term effects like infertility, and reports of myocarditis and thrombosis. Some physicians reported breakthrough infections post-vaccination, raising doubts about efficacy. Despite concerns, most healthcare professionals recommended vaccination while advising caution for individuals with chronic illnesses, oncology patients, young children, and pregnant women. However, patient reluctance persisted due to distrust, religious beliefs, and misinformation, including suspicions of financial incentives for medical professionals.

Healthcare professionals emphasized the need for improved informational resources in local languages, clearer adverse event data, and simplified brochures. They advocated for greater institutional support, social media engagement, and structured educational tools for patient concerns. Table 3 summarizes key themes from the FGDs categorized under the COM-B framework.

**Table 3:** Summary of key themes from focus group discussions categorized by the COM-B framework.

COM-B component	Theme	Illustrative quote	Behavioral driver
Capability (psychological & physical)	Lack of knowledge about vaccines	I do not really know what this vaccine is for. No one explained it properly.	Limited understanding of vaccine benefits and schedules
	Misinformation about side effects	I heard the HPV vaccine can cause infertility, so I do not want to risk it.	Fear based on rumors and misinformation
Opportunity (physical & social)	Limited access in rural areas	It’s difficult to travel to the clinic, especially in winter.	Physical barriers to healthcare access
	Influence of social networks	Most of my friends did not vaccinate their children, so I thought maybe it’s not necessary.	Peer pressure and social norms
	Language and communication barriers	The information leaflet was only in Kazakh, but I speak Russian better.	Inadequate communication from healthcare services
Motivation (reflective & automatic)	Distrust in government and healthcare system	How can we trust a system that hides information?	Institutional mistrust undermines vaccine acceptance
	Religious and personal beliefs	We believe healing comes from God, not injections.	Deep-rooted ideological resistance
	Previous negative experiences	My child had a fever after the last vaccine, so I’m afraid to continue.	Emotional response from past adverse events

#### 3.2.2 Group 2: older adult unvaccinated individuals (65+)

Older adult individuals had limited awareness of vaccine benefits and safety. Concerns included rapid vaccine development, breakthrough infections, and multiple vaccine options without clear comparative information. Some subscribed to conspiracy theories, believing vaccines were designed to “eliminate” older populations.

Participants struggled to access reliable vaccine information and feared interactions with chronic conditions. Vaccine variety caused confusion; some preferred Sputnik V for perceived safety, while others favored Pfizer for travel. Despite consulting family and doctors, most older adult individuals independently refused vaccination. Some noted that physicians stopped recommending it, and participants reported active discouragement for those over 60 years old. Logistical barriers were minimal, but participants desired fewer vaccine options and clearer medical guidance.

### 3.3 Experience of routine immunization

#### 3.3.1 Group 1: healthcare professionals

Healthcare professionals estimated vaccination coverage in their clinics at 50–95%, depending on the vaccine, but lacked regional data. They identified medical exemptions, religious and personal beliefs, and delays until one year as key barriers to vaccination. Although caregivers were aware of vaccine-preventable diseases, doctors noted higher levels of parental hesitancy towards some vaccines, particularly DPT, due to concerns about serious side effects. Some parents selectively refused vaccines, often influenced by misinformation regarding autism or neurological effects. Young mothers and migrants exhibited higher hesitancy, with some delaying vaccination to strengthen natural immunity.

Medical professionals recommended full immunization except for those with contraindications. They emphasized the need for government training on patient communication, educational materials, and media support. They also highlighted logistical challenges, including vaccine shortages and insufficient clinic space. Scheduling was generally well-organized through reminders and home visits, but unplanned vaccinations required physician approval.

#### 3.3.2 Group 3: mothers of unvaccinated children (2–2.5 years)

Mothers were aware of vaccine-preventable diseases but had limited knowledge of vaccine mechanisms. Many believed natural immunity was preferable, especially for measles. Concerns included vaccine safety, ingredient transparency, and potential health risks. Misinformation fueled hesitancy, including beliefs that vaccines caused developmental delays or that unvaccinated children were healthier. Some mothers distrusted healthcare professionals, suspecting financial incentives for vaccination. While access to clinics was not a barrier, parents preferred delaying immunization for perceived immune system development.

#### 3.3.3 Group 4: mothers of adolescent girls (9–14 years)

Mothers recognized national immunization schedules but had limited understanding of immunology. Many viewed genetic predisposition as a key disease factor and believed both vaccinated and unvaccinated individuals could contract vaccine-preventable diseases. Some rejected DTP and COVID-19 vaccines due to concerns over novelty and side effects. Hesitancy was linked to distrust in older healthcare professionals and perceptions of medical negligence. Mothers had mixed views on governmental and WHO recommendations, with social media misinformation exacerbating skepticism. Despite concerns, most adhered to routine childhood vaccination schedules unless medical exemptions applied.

Overall, vaccine hesitancy stemmed from safety concerns, misinformation, and distrust in healthcare institutions. Addressing these issues requires improved patient education, healthcare provider training, and strategic communication initiatives.

### 3.4 HPV vaccination

#### 3.4.1 Group 1: healthcare professionals

Most healthcare professionals, particularly nurses, had limited knowledge of HPV and its vaccine, often encountering misinformation about its safety and efficacy. Information sources included social media, prior pilot programs, medical conferences, and education. Concerns about infertility and the discontinued 2013 pilot program contributed to hesitancy in recommending the vaccine to patients or family members.

Post-pandemic skepticism toward new vaccines reinforced the demand for official Ministry of Health recommendations, training, and comprehensive information on vaccine safety, efficacy, composition, storage, manufacturer details, reproductive health impact, inclusion criteria, and global experiences. Preferred sources included the Ministry of Health, WHO, scientific publications, international experts, and local oncologists and gynecologists. Dissemination methods suggested included brochures, videos, and social media campaigns.

#### 3.4.2 Group 4: mothers of adolescent girls (9–14 years)

Mothers had limited awareness of cervical cancer causes, HPV, and its vaccine. Many incorrectly believed cervical cancer to be hereditary. Concerns about infertility, vaccine safety, and efficacy fueled strong opposition. Some preferred disease prevention through medication and regular screenings rather than vaccination.

Mothers relied on discussions with family members, doctors, and peers to make vaccination decisions. While medical recommendations carried weight, trust depended on provider competence and clear communication. Some younger mothers expressed distrust toward older healthcare workers.

Most mothers favored vaccination in clinics under their supervision, citing sterility concerns. Preferences varied by region: Almaty and Taldykorgan parents were open to school-based vaccination, whereas those in Pavlodar, Uralsk, and Karaganda opposed it due to concerns about inadequate medical equipment and lack of emergency response preparedness. Cleanliness, healthcare worker communication skills, and overall clinical environment influenced vaccine acceptance. Cost was not a barrier to vaccination.

## 4 Discussion

This study provides crucial insights into immunization coverage, vaccine hesitancy, and barriers to vaccine acceptance in Kazakhstan. Our findings highlight improvements in routine immunization coverage while underscoring persistent challenges related to vaccine refusal, misinformation, and public distrust in healthcare institutions. The qualitative data further revealed a complex interplay of factors influencing vaccine hesitancy, including concerns about vaccine safety, adverse effects, religious objections, and distrust in government and healthcare professionals. These issues were particularly pronounced among parents of young children and older adult individuals, who expressed skepticism over vaccine necessity and potential risks. Moreover, the study sheds light on healthcare professionals’ perspectives, emphasizing the need for better training, more accessible informational resources, and clearer communication strategies to effectively address patient concerns and misconceptions.

### 4.1 Immunization coverage and vaccine refusal trends

Immunization coverage for several vaccines improved in 2022 compared to 2020, reaching near-universal levels for DTP, PCV, MMR, and Td. However, the decline in OPV4 coverage and the increasing number of vaccine refusals indicate ongoing concerns that require targeted interventions. The failure to reach adequate immunization rates is relevant worldwide (9, 10). The rise in vaccine refusals, particularly among children under one year, suggests that hesitancy is emerging at early stages of parental decision-making. Abenova and colleagues analyzed vaccination refusal among parents and caregivers of children under 15 years of age in Kazakhstan and revealed that vaccine refusal rates had increased for 2.62 times over the observation period (11). Personal beliefs were the predominant reason for refusals, aligning with global trends (12–15) where vaccine hesitancy is often driven by misinformation and skepticism rather than logistical barriers.

### 4.2 Routine immunization perceptions

Healthcare professionals estimated routine immunization coverage at 50–95%, yet they identified persistent barriers, including medical exemptions, religious beliefs, and misinformation. According to analysis of three year data from WHO/UNICEF Joint Report Form for 2015–2017, vaccine hesitancy was observed in more than 90% of countries with one of the reasons being safety concerns (16). According to FGDs of this study, young mothers and migrant populations exhibited higher levels of hesitancy, often delaying vaccines due to concerns about natural immunity. According to studies, vaccine hesitancy among immigrant parents and families is primarily influenced by concerns and misconceptions regarding vaccine safety, insufficient awareness of vaccine-preventable diseases and immunization benefits, skepticism toward the healthcare systems and policies of host countries, language barriers, and the perception that vaccination may conflict with their religious beliefs (17, 18).

These findings underscore the importance of enhancing trust in healthcare providers and improving parental education on vaccine safety. Misinformation, distrust in healthcare institutions, and concerns about medical negligence played a significant role in hesitancy (19, 20). In this study, while some mothers accepted routine childhood vaccines, they remained skeptical of newer immunization initiatives, such as HPV vaccination. This suggests that targeted interventions must consider not only vaccine-specific concerns but also broader attitudes toward healthcare and institutional trust.

### 4.3 COVID-19 vaccination challenges

COVID-19 vaccination commenced in Kazakhstan in 2021. Prior to its implementation, Issanov and colleagues conducted a study examining knowledge and attitudes toward COVID-19 vaccination. Their findings indicated that older age and female gender were statistically significant factors associated with vaccine hesitancy (5).

Older adult unvaccinated individuals who participated in the FGDs in the present study expressed concerns regarding vaccine safety and the availability of multiple vaccine options. The presence of conspiracy theories and skepticism about the rapid development of COVID-19 vaccines further hindered uptake in this group. Similar issues have been reported in other studies (21, 22).

Healthcare professionals exhibited varying levels of knowledge and confidence in COVID-19 vaccines, with some expressing concerns about long-term safety and breakthrough infections. The presence of mixed messages, language barriers, and a lack of accessible educational materials likely contributed to their uncertainty. Similarly, the review of 35 studies on COVID-19 vaccine uptake among healthcare workers found that medical professionals had significant concerns regarding vaccine safety, efficacy and potential side effects (23).

In the present study, most healthcare professionals recommended vaccination while acknowledging patient reluctance, which was often fueled by misinformation and distrust. Addressing these issues requires enhanced public communication strategies, transparent data sharing, and clearer recommendations from healthcare professionals (24, 25).

### 4.4 HPV vaccination and public perception

HPV vaccination hesitancy was primarily driven by limited awareness and misconceptions about cervical cancer. A cross-sectional study on the knowledge and attitudes of mothers toward HPV vaccination in Kazakhstan found that only 41% of females with daughters had a positive attitude toward vaccination (26). Moreover, families with middle or upper incomes and fewer than three children were more likely to have a positive attitude toward vaccination (26). Similar findings were reported in other studies from Kazakhstan (27, 28).

The results of the present study indicate that most mothers lacked adequate knowledge of HPV transmission and vaccine efficacy, contributing to concerns about infertility and vaccine safety. The unsuccessful 2013 pilot program further reinforced skepticism (7), highlighting the importance of sustained public education efforts when introducing new vaccines. Additionally, mothers’ preference for clinic-based vaccination over school-based programs suggest that trust in healthcare infrastructure plays a critical role in vaccine acceptance.

Given these findings, effective HPV vaccine promotion should incorporate educational campaigns led by trusted sources, such as gynecologists, oncologists, and international health organizations. Disseminating accurate information through social media, brochures, and community engagement initiatives could help counter misinformation and improve public confidence in the vaccine (29, 30).

### 4.5 Strengths and limitations

This study has several strengths. First, it provides a comprehensive analysis of immunization trends, vaccine hesitancy, and barriers to vaccine acceptance in Kazakhstan, incorporating both quantitative and qualitative data. The use of qualitative methods, such as focus group discussions, allowed for a deeper exploration of individual and community-level concerns, which would not be fully captured through survey data alone. Additionally, by examining routine childhood immunization, COVID-19 vaccination, and HPV vaccination within a single study, this research offers a broad perspective on vaccine acceptance across different demographic groups and immunization programs. The findings contribute to the global discourse on vaccine hesitancy and provide valuable insights for policymakers and healthcare professionals seeking to improve vaccination strategies.

Despite these strengths, the study has certain limitations. The qualitative component, while insightful, relied on a limited number of focus group discussions, which may not fully represent the diversity of opinions across Kazakhstan. This study did not include certain groups whose perspectives may differ significantly, such as religious leaders, or vaccine-hesitant fathers, as well as representatives of ethnic minorities, individuals with disabilities, and health workers at the decision-making level. Their inclusion could have provided a broader understanding of the sociocultural and structural factors influencing vaccine acceptance and access. Additionally, self-reported data on vaccine attitudes and behaviors may be subject to social desirability bias, as participants could have provided responses they perceived as more acceptable rather than their true beliefs. Finally, while the study highlights key concerns related to vaccine acceptance, further research is needed to explore long-term trends and the effectiveness of policy changes in addressing hesitancy and improving immunization coverage.

## 5 Conclusion

This study highlights critical factors influencing vaccine hesitancy and acceptance in Kazakhstan. While routine immunization coverage has improved, significant challenges remain, particularly concerning HPV vaccination. Misinformation, distrust in healthcare institutions, and safety concerns remain key barriers to vaccine uptake. Addressing these issues requires targeted education initiatives, enhanced healthcare provider training, and strengthened institutional support. Based on our findings, we propose several targeted strategies to enhance vaccine acceptance and address hesitancy in Kazakhstan. These include community-based educational campaigns led by trusted local figures such as gynecologists and religious leaders, and the delivery of tailored communication in both Kazakh and Russian to improve vaccine literacy across diverse populations. Strengthening healthcare provider communication skills through structured training in vaccine counseling, along with increasing transparency around vaccine safety and development processes, are also critical. Furthermore, efforts should be made to address structural barriers to access, particularly in rural and underserved areas. These actionable recommendations offer a public health roadmap informed by the COM-B framework and provide practical directions for policy and program development.

## Data Availability

Data are available from the corresponding author, G.Z., upon reasonable request and with permission of the National Center for Public Health of the Republic of Kazakhstan.
