# Supplementary material for: Barriers to vaccine acceptance and immunization coverage in Kazakhstan: a mixed-methods study using the COM-B framework
Source: Front Public Health. 2025 Jun 17;13:1600363. doi: 10.3389/fpubh.2025.1600363 (PMC12209187; doi:10.3389/fpubh.2025.1600363)
Supplement: Supplementary file 1 [file Table_1.docx]

**Title:** Focus Group Guide on Vaccine Acceptance and Hesitancy in Kazakhstan

**Purpose:** To explore participants’ knowledge, attitudes, beliefs, and behavioral drivers related to routine immunization, HPV vaccination, and COVID-19 vaccination in Kazakhstan.

**Introduction speech for moderator:**

Thank you for joining today’s discussion. We are interested in your views and experiences related to vaccines and vaccination. Your insights will help us understand how to better support public health efforts in Kazakhstan. There are no right or wrong answers—we are here to listen to your perspectives

**Warm-Up Questions**

1. Can you tell us a little about yourself (e.g., age, occupation, children, where you live)?
2. What do you think about vaccination in general?

**Questions on General Vaccine Knowledge and Attitudes**

1. What vaccines do you or your children usually receive?
2. What do you think are the benefits of vaccines?
3. Are there any risks or concerns you have about vaccines?
4. Where do you get information about vaccines (e.g., doctors, internet, family)?

**Questions on Barriers and Motivations (COM-B Framework)**

***Capability:***

- Do you feel well-informed about vaccines and how they work?
- Are there things you wish you knew more about?

***Opportunity:***

- Are vaccines easily available in your community?
- Have you or someone you know faced difficulties in accessing vaccines?

***Motivation:***

- What would make you more or less likely to accept a vaccine?
- How do your personal beliefs or values influence your decision?

**Questions on Specific Vaccines**

***HPV Vaccine:***

- Have you heard of the HPV vaccine? What do you know about it?
- Would you consider vaccinating your daughter or son against HPV? Why or why not?

***COVID-19 Vaccine:***

- What are your thoughts on the COVID-19 vaccines?
- Did you or your family members get vaccinated? Why or why not

**Questions on Trust and Communication**

1. Do you trust healthcare professionals when it comes to vaccines? Why or why not?
2. How do you feel about vaccine campaigns or messages from the government or media?
3. What kind of information would make you feel more confident about vaccines?

**Closing Questions**

1. Is there anything else you’d like to share about vaccines?
2. What could be done to make vaccination easier or more acceptable for people in your community?

**Moderator speech for ending the session:**

Thank you for your participation. Your input is extremely valuable to us.
